# Radial analysis and scaling of urban land use

**DOI:** 10.1038/s41598-021-01477-y

**Published:** 2021-11-11

**Authors:** Rémi Lemoy, Geoffrey Caruso

**Affiliations:** 1 IDEES Laboratory UMR 6266 CNRS, University of Rouen, Mont-Saint Aignan, 76821 France; 2 Department of Geography, University of Luxembourg, 4366 Esch-Belval, Luxembourg; 3 Luxembourg Institute for Socio-Economic Research, 4366 Esch-Belval, Luxembourg

**Keywords:** Energy and society, Environmental impact, Sustainability

## Abstract

We determine the functional form and scaling law of radial artificial land use profiles in 300 European functional urban areas (FUAs). These profiles, starting from a fully artificial surface in the city center, decrease exponentially, the faster the smaller the city. More precisely, the characteristic decrease distance scales like the square root of total population, meaning that the artificial surface of cities is proportional to their population. This also means that the amount of artificial land per capita is independent of city size, and that larger cities are not more or less parsimonious in terms of land use than smaller ones.

## Introduction

Cities concentrate residential locations and activities of many people, and also artificial land as a consequence. This yields a main category of definitions of urban areas, which are morphological definitions, based for instance on the contiguity of urban fabric. The artificial area of cities grows with their population, but the relationship between the two quantitities is not very clear^1^, mostly because the definition of the city itself is a difficult problem. This obviously influences the relation between population and area: including or not including peripheral areas in the definition of a city has a quite important effect on its artificial area, but not as much on its population size.

Moreover, defining accurately and understanding cities is crucial because they are important elements in a variety of challenges which humanity is facing: they concentrate human activities and the associated pollutions, in a global context of climate change, scarcity of resources and pollution of the environment. They also gather in a reduced space extreme socio-economic inequalities, which makes a dangerous mix. Moreover, they are key nodes for the propagation of epidemics.

Research on urban scaling^2,3^ has been dealing with a large variety of observables, including complex social outputs like health, crime or patents. However, it seems important to ground this research in a clear understanding of some fundamental aspects of cities, in particular their physical characteristics and spatial extent. The data necessary for this endeavour is available, at the same time very detailed and covering many urban areas, and urban scaling research needs better tools to account for urban expansion. This is of course not easy, as cities are quite heterogeneous and difficult to model. Here we use the lens of radial (center-periphery) analysis, which is a nice simplifying tool for the study of the internal structure of cities. We apply it to the study of the share of land which is artificial.

With the results of previous works^4,5^, we expect a certain scaling and radial behaviour for the artificial land use profiles of European cities. We complement this research using regression methods, and characterize the mathematical form of the radial land use profiles (which we will find to be exponential), and their scaling properties. We do not propose an explanatory model, but note that a numerical model reproducing the scaling law studied here is proposed^6^. The observed scaling behaviour can be called “homothetic scaling”^4^ instead of the term “isometric scaling” originally used in biology^7^: it states that large cities present land use profiles which are similar to those of smaller cities when those are dilated proportionally (a zoom in with the right dilation factor), which is the definition of a homothetic transformation.

Although quite many works have studied the radial profiles of population density in urban areas, only few works have studied radial profiles of land use. In studies of 40 European cities^8^ and 30 Chinese urban areas^9^, the models use at least 3 or 4 parameters, which makes them difficult to compare to the present study.

One major specificity of the present work consists indeed in its parsimonious approach, studying hundreds of different cities with very simple mathematical tools, resulting in a very reduced number of model parameters. This may explain why homothetic urban scaling laws^4^ were not uncovered before, despite the amount of literature studying urban radial profiles^8–12^ (among others). We note however that the results of this study can be related to the early work of Tobler^13^ who observed close values of scaling exponents (but not to Nordbeck^4,14^).

## Data and methods

This work uses the Urban Atlas 2012 dataset developed by the Copernicus land monitoring services, which provides a precise description of land use for the nearly 700 main European cities in 2012, defined at the level of functional urban areas (FUAs, formerly denominated Larger Urban Zones LUZ). We study here, in their 2012 version, the 300 urban areas which are available both in the 2006 and 2012 versions of the Urban Atlas. Land use change in those cities between 2006 and 2012 is rather small^15^. We combine this dataset with the 1 km$^2$ population grid provided by Geostat for 2011, which covers the whole European Union. We use this population grid essentially to compute the total population of each city, as the focus of this work is mainly on land use. Our analysis is radial, studying intra-urban phenomena as functions of the distance to the city center, which is chosen as the location of the (main or historical) city hall. This definition of the center seems reasonable, as in all cases studied here it corresponds to a central location, surrounded by very artificial land use and high population densities. We note in the SI text that the precise location of the center has very little influence on our results. We then define concentric rings of width $100\sqrt{2}\simeq 141$ m around each city center, within which we average land use and distance to the center. Note that land under water is kept in this analysis (see Supplementary Information), since the data does not distinguish it from land which is simply outside of the urban area.

We focus here on the study of the artificial share of land *s*(*r*) at a given distance *r* from the city center. Since it is shown to strongly depend on the size of cities^4,5^ measured by their total population *N*, we label it $s_N(r)$. Our expectation^4,5^ for the radial evolution of these profiles is that they start at 100% in the center, since the city center (which we define as the city hall) is supposed to be on artificial land. They decrease when moving away from the center, that is when the distance *r* to the center increases and land becomes less artificial. And they should decrease faster for smaller cities. Actually, these profiles scale horizontally^4^ with the square root of the total population *N*. This can be written mathematically as $s_N(r)=s_1(r/\sqrt{N})$, with $s_1(0)=1$.

Of course, we do not expect these mathematical properties to be respected exactly: cities have local characteristics which clearly influence these land use profiles and which result in fluctuations around scaling laws^4^. However, we would like to clarify with fitting methods how well these laws are followed. In doing so, we try to follow a parsimony principle. We know that more complex models with more parameters have higher performance in terms of the goodness-of-fit, in the sense that the model is closer to the data points. However, the results of the model, which are the fitted parameters, are more difficult to study and understand: the information contained in the model is diluted among the different parameters. This can be related to the problem of overfitting, which is well-known in computer science and machine learning. Physicists usually express this problem using von Neumann’s famous quote about fitting an elephant^16^. Here we aim as a consequence to use the simplest possible model, with the least possible number of parameters but still a correct description of the dataset.

The exercise to be carried out here can be stated simply. We have 300 artificial land use radial profiles $s_N(r)$, one for each city of the dataset. As illustrated on examples in Supplementary Fig. 1, these profiles are roughly exponential, so that they look like straight lines on a semi-log graph. We want to fit them with an appropriate model respecting a parsimony principle. Our first choice considering the shape of these curves is a simple exponential fit for each city. There are however two (main) ways to carry out this exponential fit: to do a linear fit (which we denote L) of the logarithm, or to do a non-linear fit (which we denote NL) of the raw value, with a non-linear least squares fitting method.

1 $$\begin{array}{ll} {\log (s_{N} (r))} & {\sim \log (a_{N} ) - r/l_{N} ,} \\ {s_{N} (r)} & {\sim a_{N} \exp ( - r/l_{N} ),} \\ \end{array}$$

where $a_N$ is the share of artificial land in the center and $l_N$ is the characteristic distance of exponential decrease, at which the model estimates that $\exp (-1)\simeq 37\%$ of the land is artificial. In both cases, a square error is minimised, but not the same one: the linear fit of the logarithm minimises the (squared) relative error, while the non-linear fit minimises the (squared) absolute error. This can also be seen on Supplementary Fig. 1, where the non-linear fit performs better on a linear graph whereas the linear fit performs better on a semi-log graph.

## Results

### Linear and non-linear models

**Figure 1:**
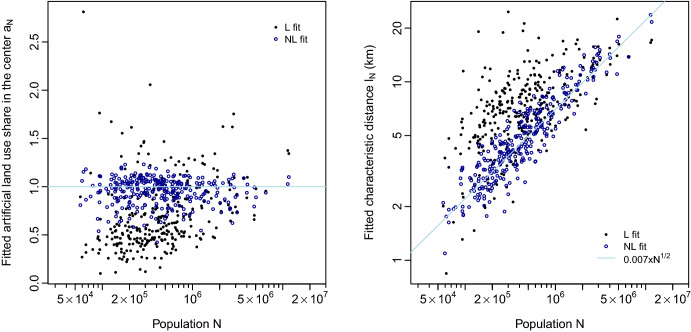
Estimated parameters of the exponential fits of artificial land use profiles, displayed as functions of the total population *N*. Predicted share $a_N$ of artificial land in the center (left) and characteristic decrease distance $l_N$ (right), for both linear and non-linear fits. The straight lines give our expectations^4^: $a_N=100$% on the left (whatever the total population), and a scaling with the square root of total population $\sqrt{N}$ on the right.

The results, which consist in two fitting parameters ($a_N$ and $l_N$) for each city, are illustrated on Fig. 1. We study how these fitted parameters evolve with total population *N*, in order to find their scaling laws. We observe that the results of the non-linear fit are closer to expectations^4^, and also more pertinent. Indeed, if we consider first the predicted share $a_N$ of artificial land in the center, the linear model gives widely dispersed values, ranging from 10% to roughly 280%. Although values above 100% are not possible in reality, a linear fit can yield such values (see Fig. 1 of SI text for an illustration), as we explain below. By comparison, the values given by the non-linear fit are much less dispersed, between 40% and 130%, and most values are concentrated around the expected 100%. Considering now the predicted characteristic decrease distance $l_N$, the non-linear fit gives again less dispersed values, which also follow much more closely the $\sqrt{N}$ scaling law^4^. In order to assess this scaling law more precisely, we subsequently fit the estimated characteristic decrease distances $l_N$ of the linear (L) and non-linear (NL) models against total population *N* (using a linear fit of the logarithms of $l_N$ and *N*), following

2 $$\begin{array}{ll} {\log (l_{N} )} & {\sim \log (l_{1} ) + \alpha \log N,} \\ {l_{N} } & {\sim l_{1} N^{\alpha } .} \\ \end{array}$$

The results are displayed in Table 1. The estimates of the linear model L follow a scaling law with an exponent around 0.3 and a low $R^2$, while the estimates of the non-linear model follow closely the $\sqrt{N}$ scaling law (which corresponds to an exponent of 0.5), with a higher $R^2$.

We see this all as pointing to the fact that the non-linear fit is more informative than the linear one, because the non-linear fit describes the city center better than the linear fit. Indeed, the non-linear fit minimises the square error of the artificial land use shares, while the linear fit minimises the square error of the logarithm of these shares. As these shares are decreasing when the distance from the city center increases, the logarithm magnifies the errors further away from the city center, putting an emphasis on the description of periurban areas. Comparatively, the non-linear fit emphasises the description of central areas. Thus, the linear model is misled by small absolute (but large relative) variations of land use in peripheral areas, which the non-linear model tends to disregard.

This might actually be an important result of this work, which probably also applies to the modelling of population density profiles. Indeed, linear models are often used in the literature^10,17,18^ , in particular for population density. The present analysis suggests to also study non-linear models.

### Two- and one-parameter models

**Figure 2:**
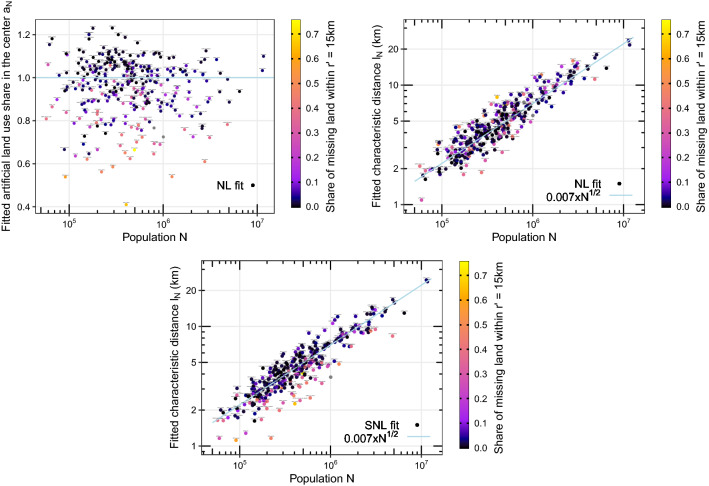
Estimated parameters of the exponential fits of artificial land use profiles, displayed as functions of the total population *N*. Predicted share $a_N$ of artificial land in the center (top left) and characteristic decrease distance $l_N$ (top right) with a two-parameter fit, and characteristic decrease distance $l_N$ with a one-parameter fit (bottom). The straight lines give expectations^4^: $a_N=100$% on the top left (whatever the total population), and a scaling with the square root of total population $\sqrt{N}$ on the top right and bottom.

Actually, even with the non-linear model, a large part of the cities are predicted to have more than 100% artificial land in their center, which is impossible. Furthermore, we observe that the city hall, which is a building hence artificial, is almost always in a very artificial area, which suggests that the city center is completely artificial. We then try to force this 100% artificial land share in the center $s_N(0)=1$, and to use a one parameter exponential fit which we call SNL (for simple non-linear model) of the artificial land profile $s_N(r)$, where the only fitting parameter is the characteristic decrease distance $l_N$: $s_N(r) \sim \exp (-r/l_N)$. The different methods are illustrated in the supplementary information. In Table 1 and on Fig. 2, we compare this new one-parameter non-linear fit SNL to the two-parameter non-linear fit NL which was the most successful so far.

**Table 1:** Results of a linear fit of the fitted (log) characteristic distance $l_N$ against the (log) total population *N*, $\log (l_N)\sim \alpha \log (N)+\log (l_1)$ for the different models: linear fit (L), two-parameter and one-parameter non-linear fits (models NL and SNL respectively) on all cities, and fits on continental cities only (models NL20 and SNL20, resp.).

Model	L	NL	SNL	NL20	SNL20
Scaling exponent $\alpha$	0.317 (0.024)	0.499 (0.013)	0.496 (0.013)	0.500 (0.012)	0.494 (0.011)
Exp(constant): $l_1$ (m)	114.4 (42.3)	7.43 (1.32)	7.04 (1.34)	7.38 (1.28)	7.69 (1.19)
Observations	293	293	293	237	237
R$^{2}$	0.372	0.845	0.826	0.877	0.895

Note that we exponentiated the second fitted coefficient in this table to obtain the distance $l_1$ (and its standard error) in meters, which is easier to interpret. $l_1$ can be seen as the characteristic size of a theoretical city^5^ housing one person ($N=1$).

We see that the two fits give similar evolutions of the characteristic size $l_N$, in agreement with the $\sqrt{N}$ scaling law^4^. However, the two models differ in particular in the way they deal with coastal cities, as illustrated by Fig. 2. We present using a color scale an indicator of the local environment of the considered city. Indeed, large bodies of water such as seas or oceans are not included within the Functional Urban Areas (FUAs), even when the city center is very close to them, which is the case for coastal cities. As a consequence, for these cities, a large part of land is missing, even close to the city center, because it is covered by a water body (in some rarer cases, land can be missing because the considered city is at the border of a country or next to a neighbouring city, which are then considered as outside of its FUA). We measure this phenomenon by the share of missing land within a disc of (rescaled) radius $r'=15$ km, where $r'=r\times \sqrt{N_{\text {London}}/N}$. This rescaling means that this disc has a radius of $r'=r=15$ km for London, the largest urban area we study. For all other urban areas, the radius is reduced proportionally to the square root of their total population *N*, according to the $\sqrt{N}$ scaling law confirmed here, so as to control for city size. For simplicity, we refer the cities with a large share of land missing close to the center as “coastal” cities, since this is the most common reason for land to be missing, while we refer other cities as “continental” cities.

On Fig. 2, we see that coastal cities are treated differently by the NL and SNL models. With the NL model, these cities are predicted to have a lower share of artificial land in the centre $a_N$ compared to continental cities. We note that this is not what happens in reality: land in the very center of coastal cities is roughly 100% artificial, as in continental cities. However, the estimated characteristic decrease distance $l_N$ of coastal cities does not really stand out. With the SNL model, the share of artificial land in the center is fixed to $a_N=100$% for all cities, and the characteristic size $l_N$ is the only parameter to optimize. In this case, we see that coastal cities have fitted characteristic sizes that stand out from cities of comparable size: this characteristic distance $l_N$ is smaller (on average) for coastal cities, all equal else. The peculiarity of coastal cities is captured by the only parameter of the SNL model, rather than being spread over the two parameters of the NL model. This shows a clear advantage of the one-parameter model: all the information extracted from the data is contained in only one parameter.

Next, we test the two models on continental cities only, and present the results in Table 1: we select only cities for which the share of missing land within a rescaled radius $r'=15$ km of the center is less than 20%, which means 237 cities out of the original 293. In this case, we call NL20 and SNL20 the log-log fits of the characteristic decrease distance $l_N$ with respect to total population *N*, for the two- and one-parameter models respectively. Both models are still compatible with the $\sqrt{N}$ scaling law, and have higher $R^2$ than the corresponding models considering all cities (NL and SNL). The increase in $R^2$ is especially noticeable for the one-parameter model. We note that this $R^2$ has a quite high value compared with those reported for urban spatial scaling laws^1^. Furthermore, the characteristic distance $l_N$ is not a usual aggregated quantity (contrary to the total surface or the total length of roads for instance): it characterises the internal structure of the city, from its center to its periphery.

### Goodness of fit

Additionally, we study for each individual city and each model two indicators of the goodness of our fits: the Bayesian Information Criterion (BIC), and the usual $R^2$—even though we note that the $R^2$ might not be as relevant for non-linear models as for linear ones^19^, we still consider it in both cases. These two indicators give actually different and complementary insights.

On one hand, the values of the BIC for all models (linear and non-linear, one- and two-parameter) decrease on average with city size, indicating that larger cities are better fitted than smaller ones. On the other hand, the values of $R^2$ change with city size only for the linear model (L), whereas the non-linear model (NL) sees no significant change of $R^2$ with city size. However, the NL model distinguishes coastal cities from continental ones: the $R^2$ of coastal cities is lower on average than the one of continental cities, indicating that coastal cities are not fitted as well as continental ones. This is especially true for the 1-parameter nonlinear model (SNL), where the $R^2$ has an average of 0.81 (standard deviation $\sigma =0.21$) for coastal cities (i.e. with more than 20% missing land within $r'=15$ km), and an average of 0.94 ($\sigma =0.05$) for “continental” cities. We note also that the loss of goodness of fit, both in terms of $R^2$ and of BIC, between the two-parameter and one-parameter non-linear models, is small. Indeed, the average (resp. median) $R^2$ for the one-parameter (SNL) model is 0.91 (resp. 0.94), whereas it is of 0.94 (resp. 0.95) for the two-parameter model (NL). Considering only “continental” cities, the values are even closer: 0.93 (resp. 0.95) for the SNL model and 0.95 (resp. 0.95) for the NL model. These values are clearly very high, which indicates that these simple models capture very well the shape of the empirical radial land use profiles.

This suggests that coastal cities are indeed special cases, which do not have a clearly exponential profile due to the land covered by water, and that the one-parameter (SNL) model detects them better. In addition, while this model is clearly more parsimonious, its description of the data is almost on par with the two-parameter model. As it also has the advantage of not overestimating the artificial share of land in the center $a_N$, we prefer it to the two-parameter model which overestimates it for some cities (where $a_N>100$%).

### Stretched exponential fit

**Figure 3:**
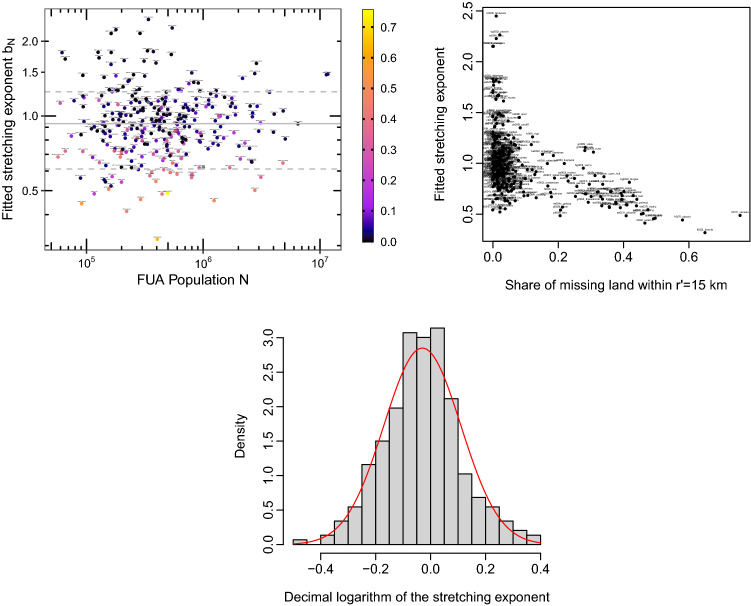
Fitted stretching exponent *b*, as a function of total population *N* (top left) and of the share of missing land within a rescaled radius $r'=15$ km (top right), indicating that coastal cities tend to have a lower value of *b*. The bottom panel shows the probability density of the decimal logarithm of this stretching exponent $\log _{10}(b)$ (black rectangles), compared with the pdf of a normal distribution of the same mean − 0.03 and standard deviation $\sigma =0.14$ (red line), used as a guide to the eye.

After having simplified our fitting method to the extreme with the one-parameter model, we can try to make it (reasonably) more complex so as to answer more precise questions regarding the shape of these artificial land profiles. In particular, we wonder whether these profiles show a global tendency to be more convex or concave than the simple exponential form which was very successful so far. In this regard, we use a stretched exponential fit $s_N(r) \sim \exp (-r^b/l_N)$, which adds a fitting parameter *b* called stretching exponent to the SNL model. The SNL model corresponds to $b=1$, which is an exponential and hence a straight line on a semi-log graph (such as the one of the right panel of Supplementary Fig. 1), with logarithmic ordinates and linear abscissae. On such a graph, $b<1$ gives a convex curve while $b>1$ gives a concave one. Using this stretched exponential model on all cities, we observe that the estimated stretching exponent *b* has a surprisingly clear lognormal distribution and seems quite independent of the total population *N*, as shown on Fig. 3. This indicates that the exponential form is very robust for the description of these land use profiles, and that fluctuations around it are rather randomly distributed. Moreover, we notice that low values of *b* often correspond to coastal cities (see Fig. 3), whose profile is usually more convex than the one of continental cities, due to the missing land covered by water. This probably explains why coastal cities are not as well fitted with an exponential fit as continental ones. We do not simply interpret high values of *b*, but notice that the cities with the most concave profiles are located in Bulgaria, Romania and the United Kingdom. In terms of goodness of fit, this stretched exponential model is slightly better than the two-parameter exponential model (NL) when averaging over all cities.

## Discussion

In this work we observe that the radial artificial land use profiles of 300 large European cities have a very clear exponential shape, with fluctuations quite randomly distributed around this exponential form. Moreover, these profiles scale with the square root of total population $\sqrt{N}$. More precisely, these artificial land profiles $s_N(r)$ can be generically described by the simple mathematical expression

3 $$\begin{aligned} s_N(r)=\exp \left( \frac{-r}{l_1 \sqrt{N}}\right) \text {, with }l_1\simeq 7\text {m.} \end{aligned}$$

Considering that this simple model has only one parameter for 300 cities, its mean and median $R^2$ over all cities are surprisingly high, with values of 0.86 and 0.92 respectively. It is striking that in this equation there is only one parameter, $l_1$ (the characteristic size of a theoretical city having just one inhabitant), which gives a scale of urban size ($l_N=\sqrt{N}l_1$), urban area ($l_N^2=Nl_1^2$) and population density ($N/l_N^2=1/l_1^2$), for a city of any size *N*. This Eq. (3) gives one of the most precise and robust (in terms of goodness of fit and number of observations) spatial scaling laws regarding urban areas which was discovered so far^1^. This scaling law also means that the artificial area of a city, which is proprotional to $l_N^2=(l_1\sqrt{N})^2\sim N$ is directly proportional to its population *N*: larger cities use just as much land per capita as smaller ones on average. Furthermore, the present analysis demonstrates the interest of non-linear fitting and of parsimonious regression approaches. Dimensional analysis issues are discussed in the Supplementary Information.

**Figure 4:**
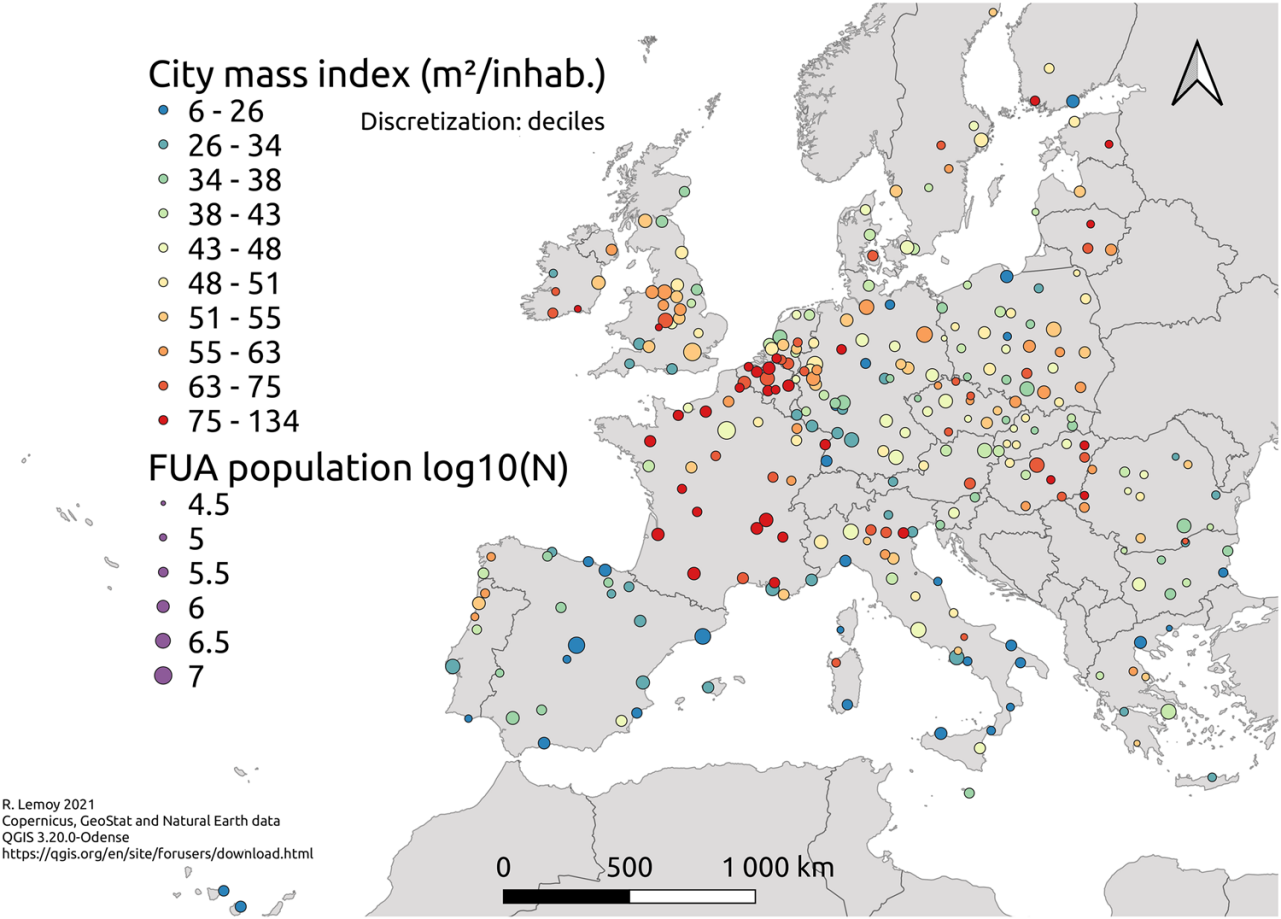
Map of the city mass index CMI, showing a clear national effect. The size of symbols indicates the decimal logarithm of FUA population *N*. The map is made using Copernicus, GeoStat and Natural Earth data with QGIS 3.20.0-Odense.

This study suggests to define a city mass index, which would be similar to the body mass index (or Quetelet index), but adapted for the social body of cities: the body mass index of humans is defined as $\text {mass}/\text {size}^2$, and the present study suggests a city mass index computed by $\text {population}/\text {size}^2$, where the size is measured by the characteristic distance $l_N$ of decrease of the artificial land use radial profile. This index gives a characteristic population density in the city’s artificial core. If $l_N$ is measured in kilometers, our analysis shows that this city mass index would have on average a value of $\langle N/l_N^2 \rangle \simeq 1/l_1^2\simeq 20,000$ inhabitants/km$^2$ in our European dataset. Higher values would correspond to compact cities, while lower values would indicate sprawled cities (not forgetting that coastal cities need to be treated separately). Actually, we prefer to use the inverse indicator $l_N^2/N$, which has more intuitive variations. It corresponds to the characteristic artificial area per capita, and would give on average $l_1^2\simeq 50$ m$^2$ in our dataset. Higher values indicate sprawled cities while lower values would indicate compact ones. This illustrates again the fact that the parameter $l_1$ provides at the same time a characteristic scale of artificial area per capita and of population density. Note that a definition of urban sprawl is suggested here: sprawled cities have a large extent in terms of artificial land use, measured by their characteristic size $l_N$, considering their population *N*.

We then define the city mass index $\text {CMI}$ of a city of total population *N* as $\text {CMI}=l_N^2/N$. The Supplementary Information shows that it is a measure of the residual of the scaling relationship (2). This index is mapped on Fig. 4.

In addition to the fact that coastal cities have low values of the CMI, as discussed before, this map shows a clear national effect: for instance, French and Belgian cities tend to have high values of the CMI, meaning that they are quite extended in terms of artificial land use compared to their total population, while Spanish ones have low values on average. This phenomenon must be studied in more detail in order to understand and improve national planning practices.

We note that this work still uses the functional urban area (FUA) as a foundation, in order to compute the total population of each city. However, we observe that there is a lot of heterogeneity between different FUAs from different countries. Fortunately, the total population size does not depend too much on the precise extent of the urban area, because the population density in the periphery is low: most of the urban population is concentrated around the core (see also the discussion in the Supplementary Information). Further work will propose new ways of defining cities based on the scaling law observed here, without a reference to FUAs. Future research should also determine whether the results obtained here can be generalized outside of Europe, and how they evolve in time. The tools developed here should be useful for the study of the evolution of urban areas, in particular regarding urban sprawl, to determine for instance whether larger cities sprawl more or less than smaller ones. This might help to understand (and maybe act on) this challenging phenomenon.

## Supplementary Information

Supplementary Information.
