# Supplementary material for: Radial analysis and scaling of urban land use
Source: Sci Rep. 2021 Nov 11;11:22044. doi: 10.1038/s41598-021-01477-y (PMC8585874; doi:10.1038/s41598-021-01477-y)
Supplement: Supplementary file 1 — Supplementary Information. [file 41598_2021_1477_MOESM1_ESM.pdf]

Supplementary information
Radial analysis and scaling of urban land use

Rémi Lemoy[1] and Geoffrey Caruso[2,3]

[1]University of Rouen, IDEES Laboratory UMR 6266 CNRS, France,

[2]University of Luxembourg, Maison des Sciences Humaines,

[3]Luxembourg Institute for Socio-Economic Research,

11 Porte des Sciences, L-4366 Esch-Belval, Luxembourg

e-mail: `remi.lemoy@univ-rouen.fr, geoffrey.caruso@uni.lu`

Keywords: radial analysis, scaling laws, urban land use

JEL: R14, R31, C55

1 Location of the city center

The city center is defined in this work as the location of the main or historical city hall, which we see as a reasonable definition. But we note here that the very precise location of the city center does not matter much at the spatial scale of the functional urban area (FUA) which we study. Indeed, the supplementary material of Lemoy and Caruso (2020) considers the influence of the location of the city center by shifting it by a few kilometers in Paris and London, and studying its influence on the obtained radial profiles. The result is that radial profiles are mostly conserved if the center is moved reasonably within the core city.

5 alternative locations are considered in London and in Paris, in addition to their main city halls. Here, we compute the influence of these shifts on the characteristic distance l_N of the (one-parameter) SNL fit, which we consider our best model. The influence is indeed very small: the coefficient of variation (relative standard deviation σ/μ, where σ is the standard deviation and μ the mean) is 0.36% for Paris and 0.16% for London.

2 Illustration of the methods

We illustrate on Figure 1 the different fitting methods used on the urban areas of London, Brussels and Namur, with population sizes of $N_{\text{London}} \simeq 12\ 000\ 000$, $N_{\text{Brussels}} \simeq 1\ 800\ 000$ and $N_{\text{Namur}} \simeq 140\ 000$ in 2006. In addition, the results of the general model given by equation (2) (main text) are also presented.

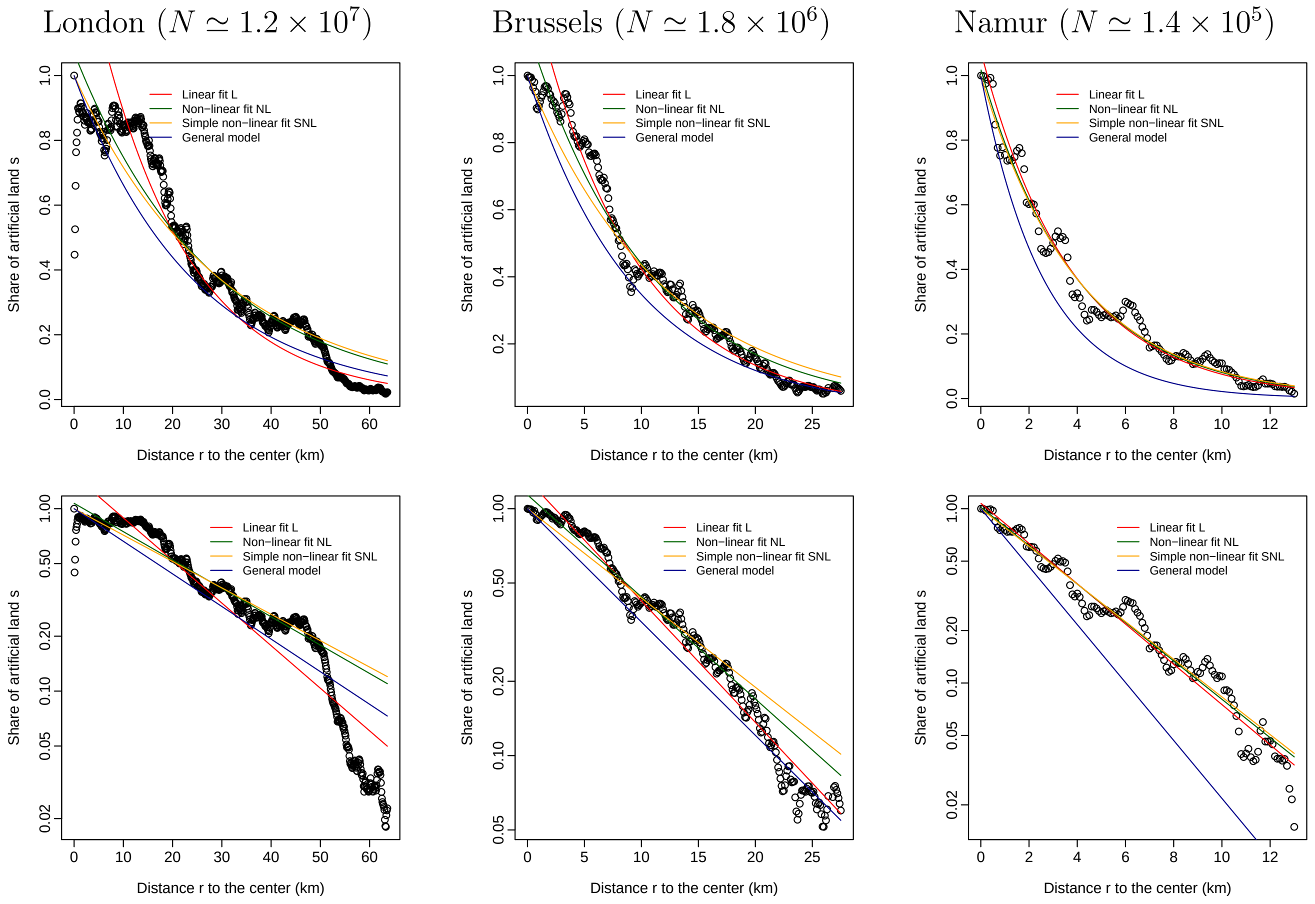

Figure 1: Illustration of the fitting methods on the cities of London (left), Brussels (middle) and Namur (right). Linear fit L, non-linear fits with two parameters (NL), with one parameter (SNL) and general model of equation (2) (main text) in linear (top) and semi-log (bottom) axes.

A parameter which might influence the goodness of fit is the resolution of the radial profiles, which determines how smooth they look. Obviously, the simple models we use correspond to very smooth curves, and the goodness of fit then improves when the empirical profiles are smoothed. With these simple models, we do not ambition to follow every local fluctuation of the radial profiles, but rather

their general shape. Consequently, it makes sense to try to smooth the curves so as to determine more precisely how successful our models are. However, we observe that this smoothing does not increase the goodness of fit measured by R^2 significantly. When smoothing the curves reasonably, the average R^2 over all cities of the NL and NL model go from 0.915 and 0.939 (without smoothing) to 0.921 and 0.944 (from 0.941 and 0.951 to 0.946 and 0.955 for the median R^2). This smoothing does not influence the scaling exponent α significantly either.

3 Coastal cities

Seeing that coastal cities are outliers in this analysis, one may wonder whether coastal cities have similar land use profiles to other cities once water is removed from the analysis. However, when performing this task we observe that coastal cities are still outliers, and that the average goodness of fit decreases when water is removed altogether. On average, coastal cities actually use more land than continental ones when water is removed, as if an excess urbanization would compensate the loss of available land due to water bodies. This is illustrated on Figure 2, which shows on its left panel the share of artificial land within a rescaled disc of radius $r' = r\sqrt{N_{\text{London}}/N} = 25$ km for all cities (this rescaling removing the effect of city size), as a function of their total population N. Coastal cities have on average a clearly lower share of artificial land than continental ones. The right panel shows that when water (or more precisely, missing land) is removed and only available land is considered, coastal cities have on average a higher share of artificial land than continental ones within a given rescaled radius. And this share of artificial available land (not covered by water) increases on average with the share of missing land (covered by water), or (equivalently) decreases with the share of available land.

4 Residuals and city mass index

The residuals of our linear regression (log-log scaling relationship (2) of the main text) can be computed as

$$\log(l_N) - \log(l_1\sqrt{N}) = \frac{1}{2}\log(\frac{l_N^2}{N}) - \log(l_1) = \frac{1}{2}\log(\text{CMI}) - \log(l_1).$$

As a consequence, the city mass index defined as $\text{CMI} = l_N^2/N$ (or rather, its logarithm) corresponds, up to some constants, to the residual of our model: it indicates how close or far a given city is from the average scaling behavior we observe.

5 Dimensional analysis

Here we note that it might seem strange in terms of dimensional analysis to relate directly a spatial quantity such as the characteristic length l_N which we compute in this work, measured in meters or

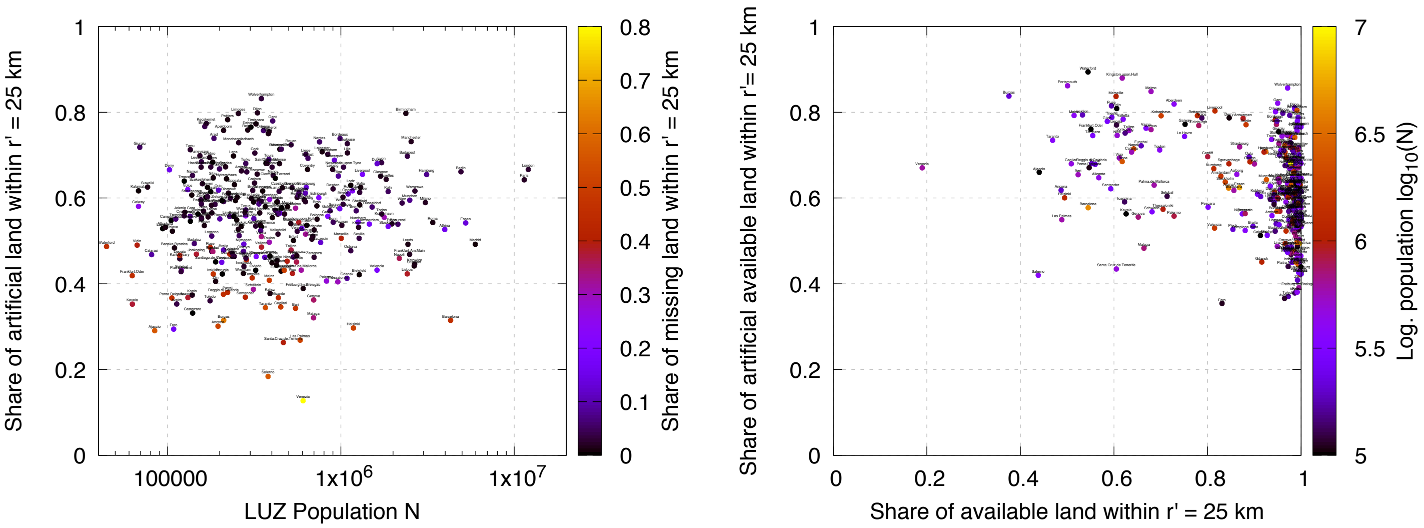

Figure 2: Share of artificial land in a rescaled radius $r' = 25$ km (left) as a function of the population N, and share of the available land which is artificial (right) as a function of the available land share of the disc of rescaled radius $r' = 25$ km.

kilometers, to the total population N, which is a count of individuals, without a dimension. However, as we argue in text, the total population N is the most reliable scaling parameter for cities, comparable to the mass of organisms for scaling studies in biology, in the sense that it is quite well defined and not too sensitive to the definition of the urban area, which is still an open question.

This can be illustrated by trying to relate this characteristic length l_N to the average population density of the FUA, as we do on Figure 3. Indeed, population density is a spatial quantity, as a ratio of total population N to total surface S of the urban area. We observe that this relation is not as clear and well defined as the one between l_N and the total population N. It is characterised by a clearly lower coefficient of determination $R^2 \simeq 0.17$.

References

Rémi Lemoy and Geoffrey Caruso. Evidence for the homothetic scaling of urban forms. *Environment and Planning B: Urban Analytics and City Science*, 47(5):870–888, 2020.

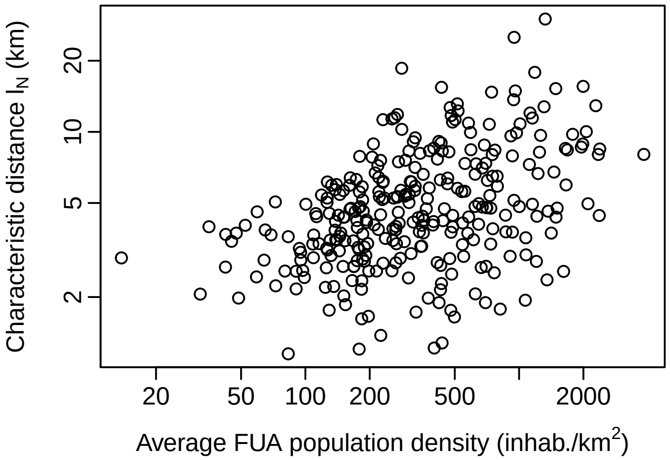

Figure 3: Relation between the characteristic length l_N and the average population density of the FUA.
